# Anxiolytic and antidepressant like effects of Zamzam water in STZ-induced diabetic rats, targeting oxidative stress, neuroinflammation, BDNF/ERK/CREP pathway with modulation of hypothalamo-pituitary–adrenal axis

**DOI:** 10.3389/fnins.2023.1265134

**Published:** 2023-12-01

**Authors:** Medhat Taha, Mohamed Ezzat Mahmoud, Abdullah G. Al-Kushi, Anas Sarhan, Omer Abdelbagi, Tourki A. S. Baokbah, Omar Babateen, Ibrahim El-Shenbaby, Naeem F. Qusty, Sara T. Elazab

**Affiliations:** ^1^Department of Anatomy and Embryology, Faculty of Medicine, Mansoura University, Mansoura, Egypt; ^2^Department of Anatomy, Al-Qunfudah Medical College, Umm Al-Qura University, Al-Qunfudah, Saudi Arabia; ^3^Histology Department, Damietta Faculty of Medicine, Al-Azhar University, Damietta, Egypt; ^4^Department of Human Anatomy, Faculty of Medicine, Umm Al-Qura University, Makkah, Makkah, Saudi Arabia; ^5^Department of Internal Medicine, College of Medicine, Umm Al-Qura University, Makkah, Saudi Arabia; ^6^Department of Pathology, Qunfudah Faculty of Medicine, Umm-Al-Qura University, Al-Qunfudah, Saudi Arabia; ^7^Department of Medical Emergency Services, College of Health Sciences-AlQunfudah, Umm Al-Qura University, Al-Qunfudah, Saudi Arabia; ^8^Department of Physiology, Faculty of Medicine, Umm Al-Qura University, Makkah, Makkah, Saudi Arabia; ^9^Clinical Pharmacology Department, Faculty of Medicine, Mansoura University, Mansoura, Egypt; ^10^Medical Laboratories Department, Faculty of Applied Medical Sciences, Umm Al-Qura University, Makkah, Saudi Arabia; ^11^Department of Pharmacology, Faculty of Veterinary Medicine, Mansoura University, Mansoura, Egypt

**Keywords:** anxiety, depression, Zamzam water, diabetes, oxidative stress, neuroinflammation

## Abstract

**Introduction:**

Recent studies have reported a strong relationship between diabetes and anxiety- and depression-like behaviors; however, there is a lack of information on the underlying pathophysiology. Alkaline Zamzam water (ZW), which is rich in several trace elements, has neuroprotective properties. This study aimed to investigate the anxiolytic and antidepressant effects of ZW against diabetes-induced behavioral changes and shed light on the possible underlying mechanisms.

**Methods:**

Forty-eight rats were divided into four experimental groups (*n* = 12): group I (control group), group II (Zamzam water group), group III (diabetic group), and group IV (diabetic + Zamzam water group). Diabetes was induced by an intraperitoneal injection of 60 mg/kg streptozotocin (STZ). At the end of the experiment, the forced swimming test (FST) was used to assess depression-like effects. The elevated plus maze test (EPMT) and open field test (OFT) were performed to evaluate anxiety-like behavior. Blood levels of the hypothalamic–pituitary–adrenal (HPA) axis were measured, and prefrontal cortex and hippocampal tissue samples were removed for histological, immunohistochemical, ELISA, and Q-PCR analyses.

**Results:**

ZW significantly decreased the immobility time in the FST, indicating an antidepressant effect (*p* < 0.001). Additionally, ZW significantly improved the OFT and open field entry (OFE) percentages in the EPMT, increasing center crossing and decreasing grooming and fecal boli in the OFT. This indicated an anxiolytic-like effect in diabetic rats with histological improvement. Interestingly, ZW significantly increased prefrontal cortical and hippocampal levels of antioxidant enzymes and the Nrf2/HO-1 pathway. It also modulated the HPA axis by increasing cortisol and corticotropin-releasing hormone (CRH) levels, with a decrease in ACTH and an increase in monoamine neurotransmitters. Furthermore, diabetic rats that received ZW showed a decrease in the inflammatory markers TNF-α and GFAP by immunohistochemistry and in the mRNA levels of NFκB, IL-1β, and IL6. In addition, ZW downregulated the expression of the BDNF/ERK2/CREP pathway.

**Conclusion:**

Our results suggested a neuroprotective effect of ZW against diabetes-induced anxiety- and depression-like behaviors and explored the underlying mechanisms. These findings suggest a promising therapeutic strategy for patients with diabetes who experience anxiety and depression.

## 1 Introduction

Diabetes mellitus (DM) is one of the most common metabolic disorders in the world. Its prevalence varies among countries, with 415 million people living with diabetes globally. This number is expected to increase to 642 million by 2040 (Ogurtsova et al., 2017). Unfortunately, diabetes is commonly associated with anxiety and depression due to hormonal and neurochemical changes (Collins et al., 2009; Menezes Zanoveli et al., 2016). The incidence of anxiety and depression in diabetic patients is 30 to 40% higher than that of healthy individuals (Roy and Lloyd, 2012). The role of hyperglycemia in the pathophysiology of psychological comorbidities remains unclear (Wrighten et al., 2009). Diabetes mellitus involves several mechanisms in the pathogenesis of anxiety- and depression-like states. These include oxidative stress and free radical formation, pro-inflammatory cytokine generation, neurotransmitter dysregulation, modification of the hypothalamic–pituitary–adrenal (HPA) axis function, and synaptic plasticity impairment in different brain areas, such as the hippocampus and prefrontal cortex (Schiepers et al., 2005; Beauquis et al., 2010; de Morais et al., 2014). Thus, treatments with antioxidant and anti-inflammatory properties should be examined to determine their role in minimizing the symptoms of anxiety and depression in patients with diabetes.

Brain-derived neurotrophic factor (BDNF) is considered an essential peptide for axon growth, neuronal survival, and synaptic plasticity and has an up-regulatory role in the extracellular signal-regulated kinase (ERK) pathway in depression (Autry and Monteggia, 2012). Several studies have reported that BDNF and its receptor, tropomyosin-related kinase B (TrkB), are involved in the pathogenesis and treatment of depression (Chen et al., 2001). ERK is an intracellular signal that responds to stress and plays a vital role in ERK processing (Gourley et al., 2008). ERK plays a crucial role in the phosphorylation of cyclic adenosine monophosphate response element-binding protein (CREP; Gourley et al., 2008). CREP is involved in stress-dependent emotional abnormalities (Wallace et al., 2009). The mechanism of action of several antidepressants is based on the upregulation of CREP phosphorylation (Blendy, 2006).

Zamzam water (ZW) is a well-known alkaline water that differs from the physical and chemical properties of other types of water. Physical analysis of ZW revealed its alkaline nature, and its content includes several trace elements, such as selenium, zinc, and magnesium, which contribute to forming antioxidant enzymes (Taha et al., 2023a,b). A previous study explored its reductive effect on the level of HbA1c-glycated hemoglobin in diabetic patients after 2 months of use (Bamosa et al., 2013). Another experimental study has documented its ameliorative antioxidant effects on aging (Hofer et al., 2008) and gentamicin-induced stress (Abdullah et al., 2012). Therefore, this study aimed to examine the impact of ZW on STZ-induced anxiety and depression and the possible underlying neuroprotective mechanisms.

## 2 Materials and methods

### 2.1 Animals

Forty-eight male albino rats (200–230 g) were purchased from the Faculty of Veterinary Medicine at Zagazig University. The rats in the different experimental groups were housed in plastic cages with dimensions of 41 × 32 × 16.5 cm and a controlled temperature of 22 ± 2°C under a 12 h day/12 h night cycle. The animals in the experimental groups I and III had *ad libitum* access to food and water, and groups II and IV consumed ZW. All the experiments were conducted according to the ethical guidelines of the Animal Research Committee of MU-ACUC, Mansoura University (Code: VM.R.22.12.37). The rats were acclimatized for 2 weeks before the experiments to accommodate the experimental environment.

### 2.2 Induction of diabetes

To induce type 1 diabetes mellitus, rats in groups III and IV were injected intraperitoneally with 60 mg/kg of STZ (Santa Cruz Biotechnology Inc., United States) dissolved in citrate buffer (10 mM, pH 4.5; Pereira et al., 2018). Elevated blood sugar levels were confirmed 72 h after STZ injection by placing peripheral blood samples from rat tail veins on test strips impregnated with glucose oxidase (Accu-Check Active^TM^; Roche). Rats with a blood sugar level above 250 mg/dL were considered hyperglycemic and were retained in the behavioral tests, whereas rats with blood sugar levels less than 250 mg/dL were excluded from the study (Gandhi and Sasikumar, 2012). Rats in the different groups were observed throughout the study, and their weight and blood sugar levels were measured.

### 2.3 Experimental design

The four groups of 12 rats were categorized as follows: Group I (control group): non-diabetic control rats that were administered tap water and citrate buffer (10 mM, pH 4.5); group II (ZW group): control non-diabetic rats that were administered ZW at a dose of 100 mg per cage. After intraperitoneal injection of 60 mg/kg STZ, diabetic rats were divided into groups III and IV: group III (diabetic group), control diabetic rats that received tap water; group IV Diabetic + ZW), diabetic rats that received 100 mL of ZW per cage/day. Behavioral tests were performed from days 28 to 30 after diabetic induction. Anxiety-like behavior was assessed using two common behavioral tests: the EPMT and open-field arena. Depression-like behavior was assessed using the forced swimming test (FST). The Noldus Ethovision XT video tracking system was used to record and evaluate the results of behavioral tests.

### 2.4 Behavioral tests

#### 2.4.1 Forced swimming test

FST was performed in a Plexiglas cylinder with a height of 46 cm and an internal diameter of 21 cm, and the cylinder was filled with water to a depth of 30 cm (23 ± 1°C). The water was changed between swim sessions. In the first pre-test session, the rats were left in the water for 15 min. The test session was performed 24 h later by placing the rats in a cylinder for 5 min and recording their immobility behavior. Immobility can be defined as the cessation of fighting, with rats floating and keeping their heads above the water. Immobility was considered an indicator of depression. Video recordings were analyzed by a blinded examiner (Zhu et al., 2013).

#### 2.4.2 Elevated plus maze test

The EPMT is one of the most well-known tests used to examine anxiety-like behaviors in rodents. The maze used in the test was cross-shaped, with two open arms (50 cm × 10 cm) and two closed arms (50 cm × 10 cm × 40 cm) elevated 50 cm above the ground. The rats in the different experimental groups were placed in the center of the maze and left for 5 min with free movement; the test session was video recorded. The number and time of the entry of the rats into the open and closed arms with four paws were recorded. The percentage of entries into the open arm was calculated using the following equation: [(ratio of entries into the open arms to total entries) × 100] The percentage of time spent in the open arm was calculated as follows: [(ratio of time spent in the open arms to the total time spent in any arm) × 100]. The total arm entries were an indicator of locomotor activity (Kosari-Nasab et al., 2013; Farajdokht et al., 2017).

#### 2.4.3 Open field test

The open field test is a well-known anxiety test. It was performed using an open field apparatus that formed an arena (50 × 50 × 40 cm) with a brown floor divided into nine squares and black plywood walls. Each rat from each experimental group was placed in the center of the apparatus for 5 min and allowed to move freely to explore a new environment. The behavior of all rats, including the time spent in the center, grooming time, travel distance, and number of fecal boli, was recorded and analyzed by a blinded observer (Mosaferi et al., 2015).

### 2.5 Brain samples

Two hours after the last behavioral experiment, the rats were humanely euthanized by cervical dislocation under deep sedation induced by an injection of 90 mg/kg ketamine and 15 mg/kg xylazine. The brains were then removed. The prefrontal cortex and hippocampus were removed, and some tissues were fixed in 10% neutral-buffered formalin and placed in wax for histopathological examination. The rest of the brain tissue was frozen in liquid nitrogen and stored at −80°C for further analysis. The brain samples were homogenized in a 1:10 dilution of potassium phosphate buffer (0.1 M, pH 6.5). Oxidative stress indicators were assessed using tissue homogenates.

### 2.6 Blood sampling for assessment of the HPA axis

After behavioral testing for 24 h, the rats in the different experimental groups were sacrificed by cervical dislocation, and a blood sample was collected from the retroorbital venous plexus. The serum was separated by centrifugation at 1000 × g for a quarter-hour. Subsequently, the serum was stored at −80°C to analyze cortisol, ACTH, CRH, TNF-α, CRP and total antioxidant capacity.

### 2.7 Biochemical parameters

Commercial kits (Biodiagnostics Co., Cairo, Egypt) were used with the aid of a spectrometer assistant according to the manufacturer’s instructions. To measure the levels of malondialdehyde (MDA), and the antioxidant enzymes; superoxide dismutase (SOD), catalase (CAT), and glutathione (GSH) were measured in the brain tissue supernatant.

### 2.8 Histological and immunohistological evaluation

The brain samples were preserved in 10% buffered formalin before immersion in paraffin blocks for histopathological examination. The coronal serial sections (5 μm) of the hippocampus and prefrontal cortex were dehydrated with ascending grades of ethanol and stained with hematoxylin and eosin (H&E). For immunohistochemical examination, 5 μm paraffin sections were rehydrated with descending scales of ethanol (100, 95, and 70% ethanol) and then washed with distilled water for 5 min. The sections were rinsed with protein-buffered saline (PBS). To block endogenous peroxidase, sections were treated with 0.1% hydrogen peroxidase (H_2_O_2_) for 30 min and rinsed again with PBS. The sections were then incubated with a blocking solution (10% normal goat serum) for 1 h at room temperature. The sections were incubated with primary antibodies for TNF-α (anti-TNF-α rabbit, ab6671, Abcam), GFAP (anti-GFAP rabbit pAb, GB11096, Servicebio), HO-1 (anti-HO-1, ab189491, Abcam), and active caspase-3 (anti-active caspase-3 rabbit pAb, GB11532, Servicebio) for 60 min at room temperature. Subsequently, the sections were rinsed with PBS. The sections were then incubated with secondary antibodies for 20 min at room temperature and rinsed with PBS. “Streptavidin-Horseradish peroxidase” solution enzyme conjugates were applied for 10 min. The conjugated secondary antibody sites were visualized using 3,3-diaminobenzoic acid (DAB), washed with PBS, and counterstained with hematoxylin. Sections of the prefrontal cortex and hippocampus were photographed using a digital camera (Olympus) by a blinded expert pathologist at the Pathology Department, Veterinary Medicine College, Mansoura University. The number of immunostained cells in the fields taken from at least three rats was counted using ImageJ software and averaged per field for each animal.

### 2.9 Enzyme-linked immunosorbent assay examination

The supernatant was used to measure serotonin, dopamine, norepinephrine, tropomyosin receptor kinase B (TrkB receptor), phospho-extracellular signal-regulated kinase 1/2 (p-ERK1/2), rat phosphatocytic AMP response element binding protein (p-CREB), and BDNF levels using commercial ELISA kits (Cat# E-EL-R1140, DOU39-K01, NOU39-K01 0, NBP2-76777, EMS2ERKP, MBS7255484, and SEA011Ra), while serum levels of total antioxidant capacity, CRP, and TNF-α were assessed by using commercial ELISA kits (Cat# ab65329, MBS494066, and KHC3011) according to the manufacturer’s instructions.

### 2.10 RT-PCR assessment

Total RNA was extracted from brain tissue using phenol and guanidinium isocyanate (Trizol reagent 15,596,026, Life Technologies, United States). Spectrophotometric techniques were used to determine the purity and quantity of the RNA at wavelengths of 260–280 nm. Following the manufacturer’s instructions, 1 μgm of RNA was converted into single-strand paired DNA using the Quali-Tect Reverse Transcription Kit (Qiagen). Table 1 shows the primers used for interleukin-6 (IL6), interleukin-1 beta (IL-1β), nuclear factor erythroid 2-related factor 2 (Nrf2), heme oxygenase-1 (Ho-1), and reference control (GAPDH) genes. Universal SYBR Green qRT-PCR was used to conduct a real-time polymerase chain reaction (PCR). Denaturation was performed according to standard procedures. Rotor-gene Q automatically analyzed the data and determined the upper limit of housekeeping genes.

**Table 1:** Primer sequences for the examined genes.

Gene	Primer sequence
IL-6	F: 5’-GTCTTCTGGAGTTCCGTTTCT-3′ R: 5’-GGGTTTCAGTATTGCTCTGAATG-3’
IL-1β	F: 5’-CGTGGGATGATGACGACCTG-3′ R: 5’-TGGGTGTGCCGTCTTTCATC-3’
Nrf2	F: 5’-AGGACATGGAGCAAGTTTGG-3′ R: 5’-TTGCCCTAAGCTCATCTCGT-3’
NFκB	F: 5’-GTCTCAAACCAAACAGCCTCAC-3′ R: 5’-CAGTGTCTTCCTCGACATGGAT-3’
GAPDH	F: 5’-GGTGTGAACCACGAGAAATATGAC-3 R: 5’-TCATGAGCCCTTCCACAATG-3’

### 2.11 Statistical analysis

The findings were presented as mean ± standard deviation, and an ANOVA and a Tukey–Kramer *post-hoc* test were performed using GraphPad Prism software (version 8.0; La Jolla, CA, United States). Statistical significance was set at *p* < 0.05.

## 3 Results

### 3.1 Impact of Zamzam water on behavioral parameters (antidepressant-like behavior and anxiety-like behavior) of STZ-induced diabetic rats

**Figure 1:**
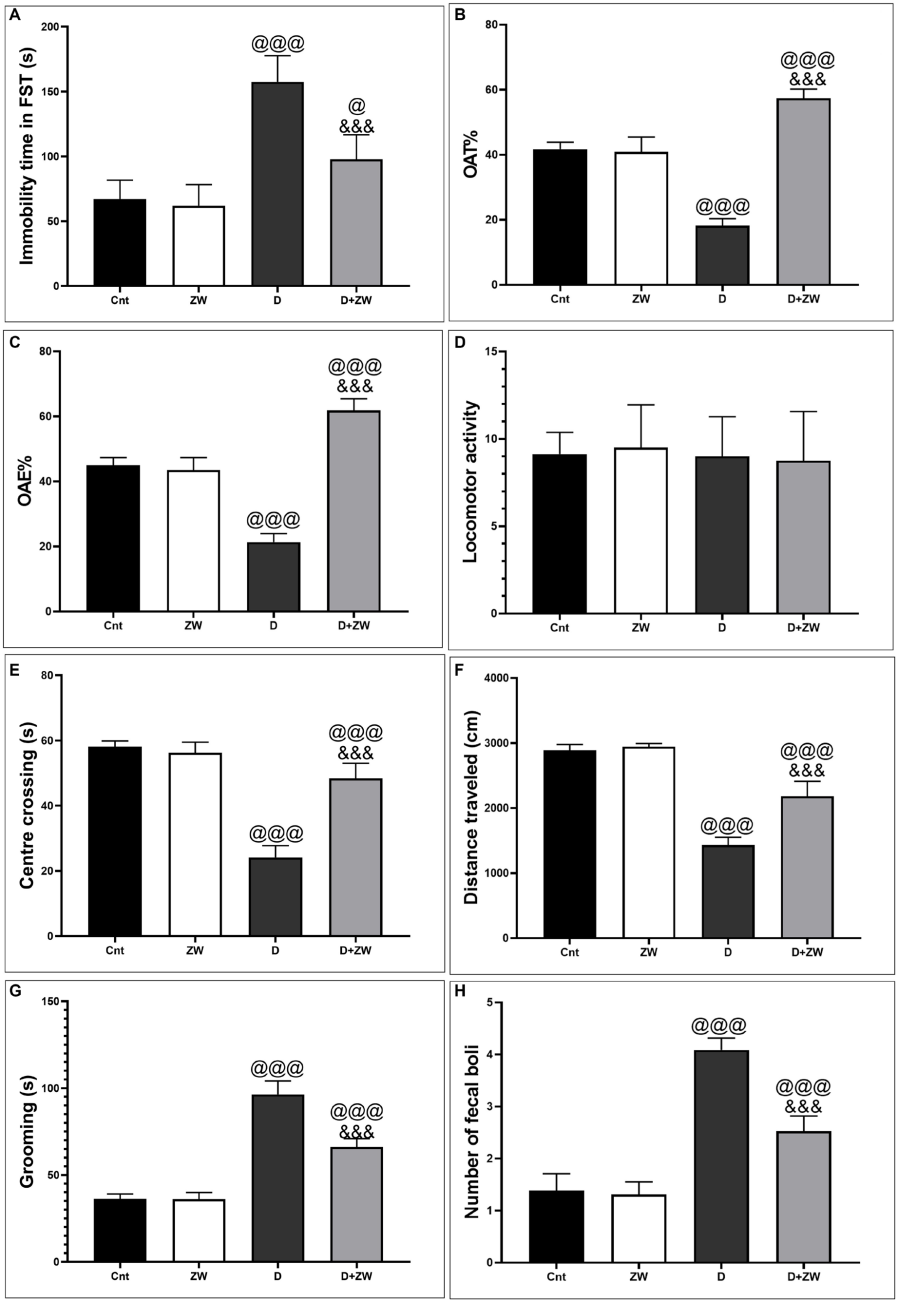
Impact of ZW on antidepressant-like behavior in FST. **(A)** Immobility time and anxiety-like behavior tests 1. Elevated plus-maze **(B)** open-arm time%, **(C)** open-arm entry%, and **(D)** locomotor activity 2. Open field test: **(E)** Center of crossing; **(F)** Distance traveled; **(G)** Grooming; and **(H)** Number of fecal boli between different experimental groups. Data were expressed as mean ± SD. ^@@@^*p* < 0.001 vs. the control group; ^&&&^*p* < 0.001 vs. the diabetic group.

As shown in Figure 1A, the immobility time in FSTs increased significantly (*p* < 0.001) in diabetic rats by 234.6% compared with control rats, whereas treatment with ZW significantly (*p* < 0.001) decreased the immobility time by 59.62% compared with the diabetic group, indicating that ZW has a positive impact on minimizing depression-like behavior. Likewise, ZW significantly *p* < 0.001 decreased anxiety-like behavior and elevated maze test scores by increasing the open arm time% and open arm entry% (Figures 1B,C) by 314.4 and 289.4%, respectively, compared with the diabetic group. Additionally, in the OFT, our findings revealed that ZW significantly *p* < 0.001 increased the center-crossing time by 200.4% (Figure 1E), the distance traveled by 152.4% (Figure 1F), and the time spent grooming by 68.8% (Figure 1G), and the number of fecal emboli was 61.7% of that in diabetic rats (Figure 1H). However, there was no significant difference in locomotor activity between the different experimental groups (Figure 1D). No significant differences were found between the control and ZW groups.

### 3.2 Effect of Zamzam water on the hypothalamic–pituitary–adrenal axis pathway

**Figure 2:**
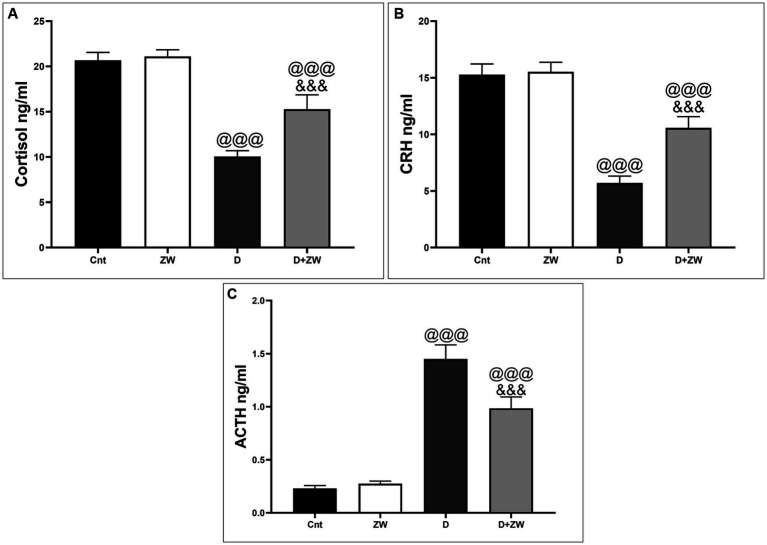
Effect of ZW on serum HPA axis hormone levels. **(A)** Cortisol, **(B)** CRH, and **(C)** ACTH. Data were expressed as mean ± SD. ^@@@^*p* < 0.001 vs. the control group; ^&&&^*p* < 0.001 vs. the diabetic group.

As shown in Figure 2, serum analysis of hormones (HPT axis) in diabetic rats revealed a significant (*p* < 0.001) decrease in the levels of cortisol and corticotropin-releasing hormone (CTH) by 48.5 and 37.3%, respectively (Figures 2A,B), and an increase in adrenocorticotropic hormone (ACTH) levels by 630% (Figure 2C) compared with that of the control group. In contrast, ZW-treated diabetic rats showed a significant increase (*p* < 0.001) in the serum levels of cortisol and CRH of 152 and 185.2%, respectively, with a decrease in ACTH of 67.5%, compared to diabetic rats. These changes confirmed the regulatory role of ZW in HPA axis diabetic rats.

**Figure 3:**
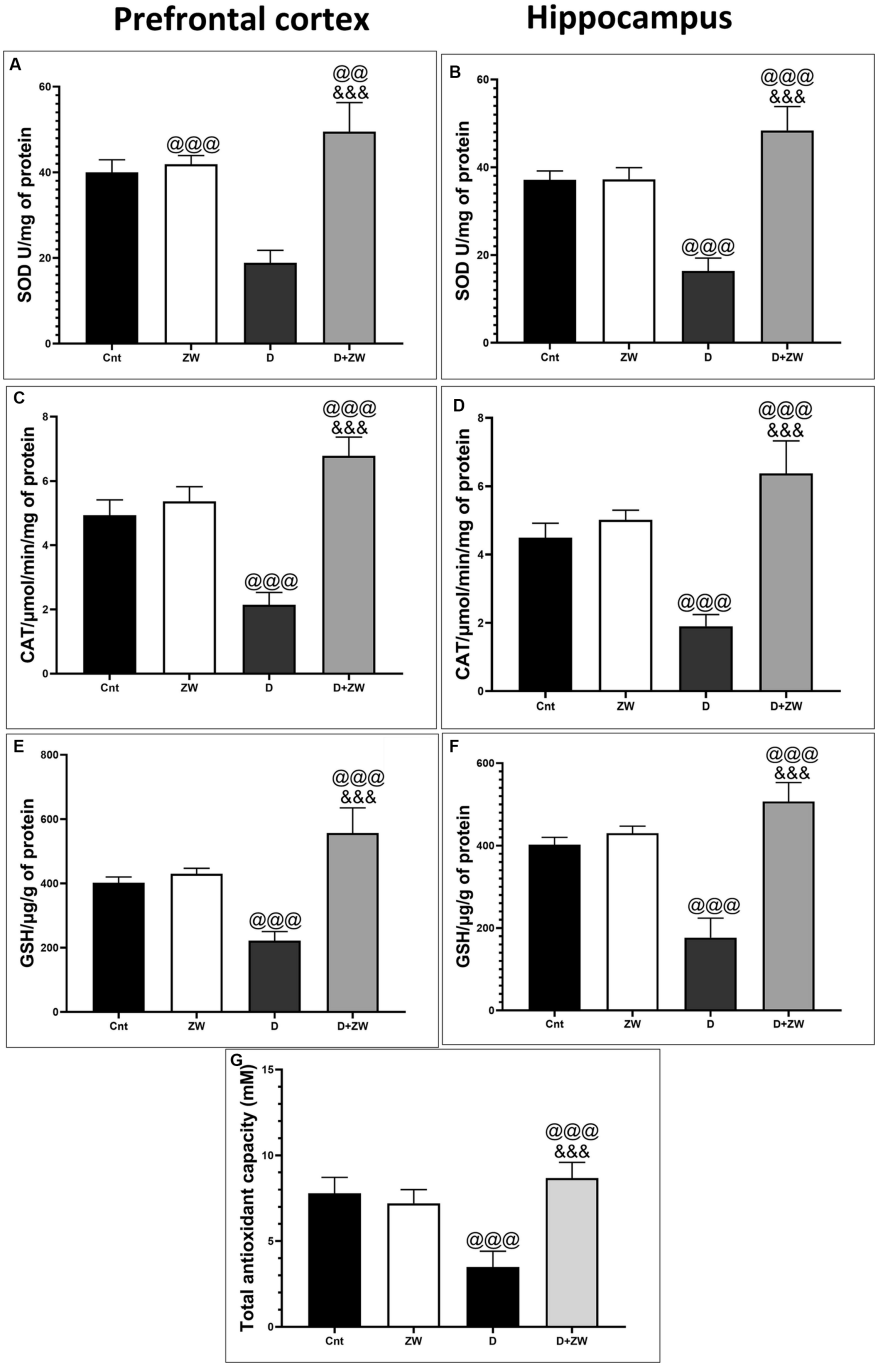
Effect of ZW on SOD, CAT, and GSH levels in the prefrontal cortex **(A,C,E)**, hippocampus **(B,D,F)** and serum level of Total antioxidant capacity **(G)** of diabetic rats. Data were expressed as mean ± SD. ^@@@^*p* < 0.001, ^@@^*p* < 0.01 vs. the control group; ^&&&^*p* < 0.001 vs. the diabetic group.

### 3.3 Effect of Zamzam water on superoxide dismutase, catalase, and glutathione in the prefrontal cortex and hippocampus, and serum level of total antioxidant capacity of STZ-induced diabetic rats

**Figure 4:**
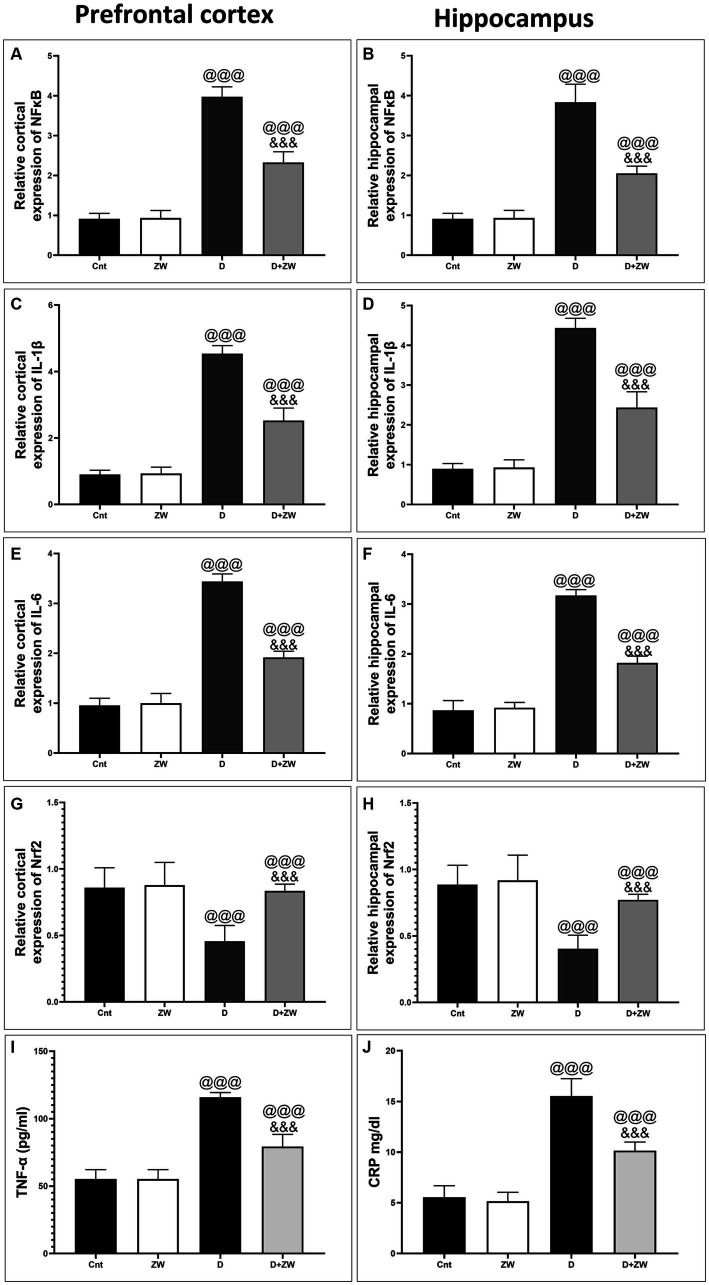
Effects of ZW on relative gene expression in the prefrontal cortex and hippocampus. **(A,B)** NFκB **(C,D)** IL-1β **(E,F)** IL-6 **(G,H)** Nrf2, and serum level of TNF-α, CRP **(I,J)**. Data were expressed as mean ± SD. ^@@@^*p* < 0.001 vs. the control group; ^&&&^*p* < 0.001 vs. the diabetic group.

**Figure 5:**
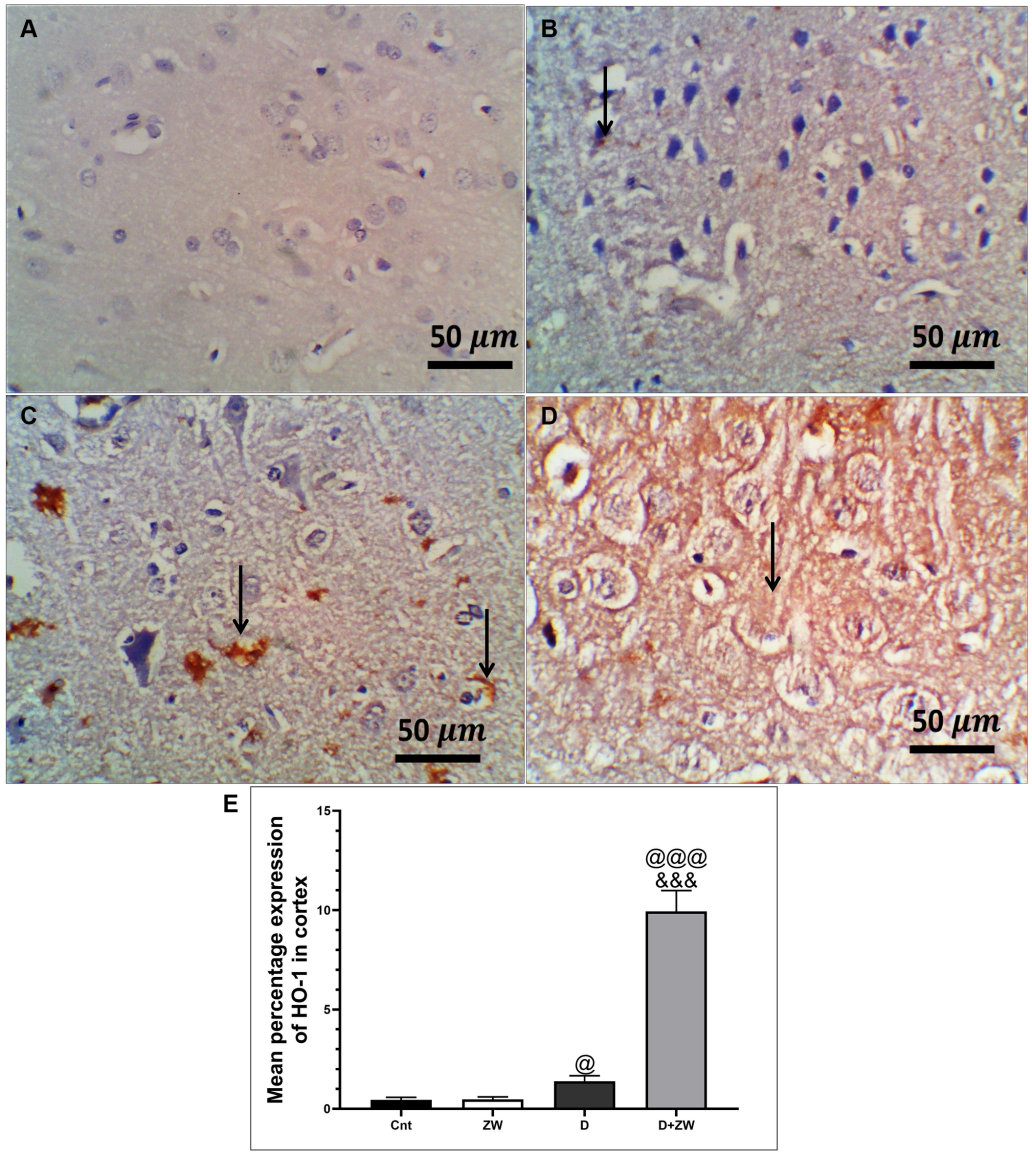
Representative photomicrographs of Ho-1 expression in the prefrontal cortex of different treatment groups. **(A)** The control group showed no Ho-1 expression in the cortex or the hippocampus. **(B)** The Zamzam group showed occasional expression of Ho-1 in the neurophil of the cortex. **(C)**The diabetic group demonstrated low cortical expression. **(D)** The D + ZW group exhibited diffuse and strong expression of Ho-1 in the neurophiles and neurons of the cortex. The thin arrows indicate positive immunostaining. **(E)** Data were expressed as mean ± SD. ^@@^*p* < 0.01, ^@@@^*p* < 0.001 vs. the control group; ^&&&^*p* < 0.001 vs. the diabetic group. The image magnification was 400x.

**Figure 6:**
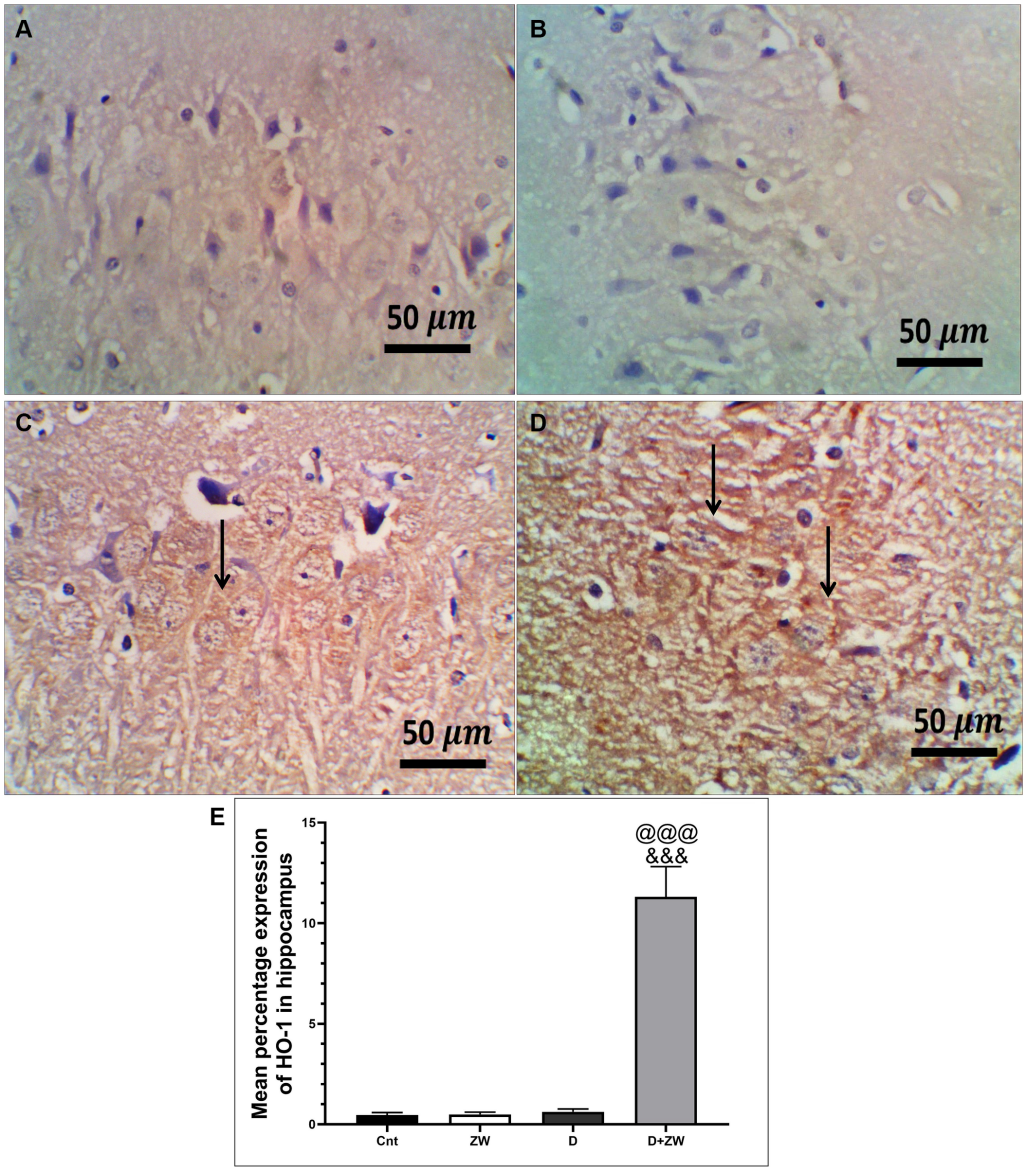
Representative photomicrographs of Ho-1 expression in the hippocampus region CA3 of the different treatment groups. **(A)** The hippocampal control group showed no Ho-1 expression in the cortex or the hippocampus. **(B)** The Zamzam group demonstrated an occasional lack of Ho-1 in the neurons of the hippocampus. **(C)** The diabetic group showed mild, faint, immunopositive neurons in the pyramidal layers of the CA3. **(D)** The D + ZW group exhibited diffuse and strong expression of Ho-1 in the neurophiles and neurons of the hippocampus. The thin arrows indicate positive immunostaining. The image magnification was 400x. **(E)** Data were expressed as mean ± SD. ^@@^*p* < 0.01; ^@@@^*p* < 0.001 vs. the control group; ^&&&^*p* < 0.001 vs. the diabetic group. Magnification = 50x, and inset = 400x.

Diabetic rats receiving ZW showed a significant increase (*p* < 0.001) in the activity of the antioxidant enzymes SOD, CAT, and GSH by 262.1, 316.8, and 250.8%, respectively, in prefrontal cortex tissue homogenates (Figures 3A,C,E) and 295.5, 335.2, and 286.7% in hippocampal homogenates (Figures 3B,D,F) compared with diabetic rats. Furthermore, ZW significantly upregulated (*p* < 0.001) the Nrf2/HO-1 pathway by increasing Nrf2 mRNA expression by 184.4 and 192.5% (Figures 4G,H) and immunoexpression of antioxidant response element (ARE) HO-1 by 719.5 and 1824%, respectively (Figures 5D, 6D) in the prefrontal cortex and hippocampus, respectively, compared with the diabetic group (Figures 5C, 6C). Regarding the peripheral effect of ZW on diabetes-induced oxidative insult, diabetic group received ZW showed a significant (*p* < 0.001) increase in the serum level of total antioxidant capacity by 249.1% in comparison to diabetic rats (Figure 3G). The positive effect of ZW on the Nrf2/HO-1 pathway explained its enhanced antioxidant enzyme activity and properties.

### 3.4 Influence of Zamzam water on the histological structure of the prefrontal cortex and CA3 of the hippocampus of STZ-induced diabetic rats

**Figure 7:**
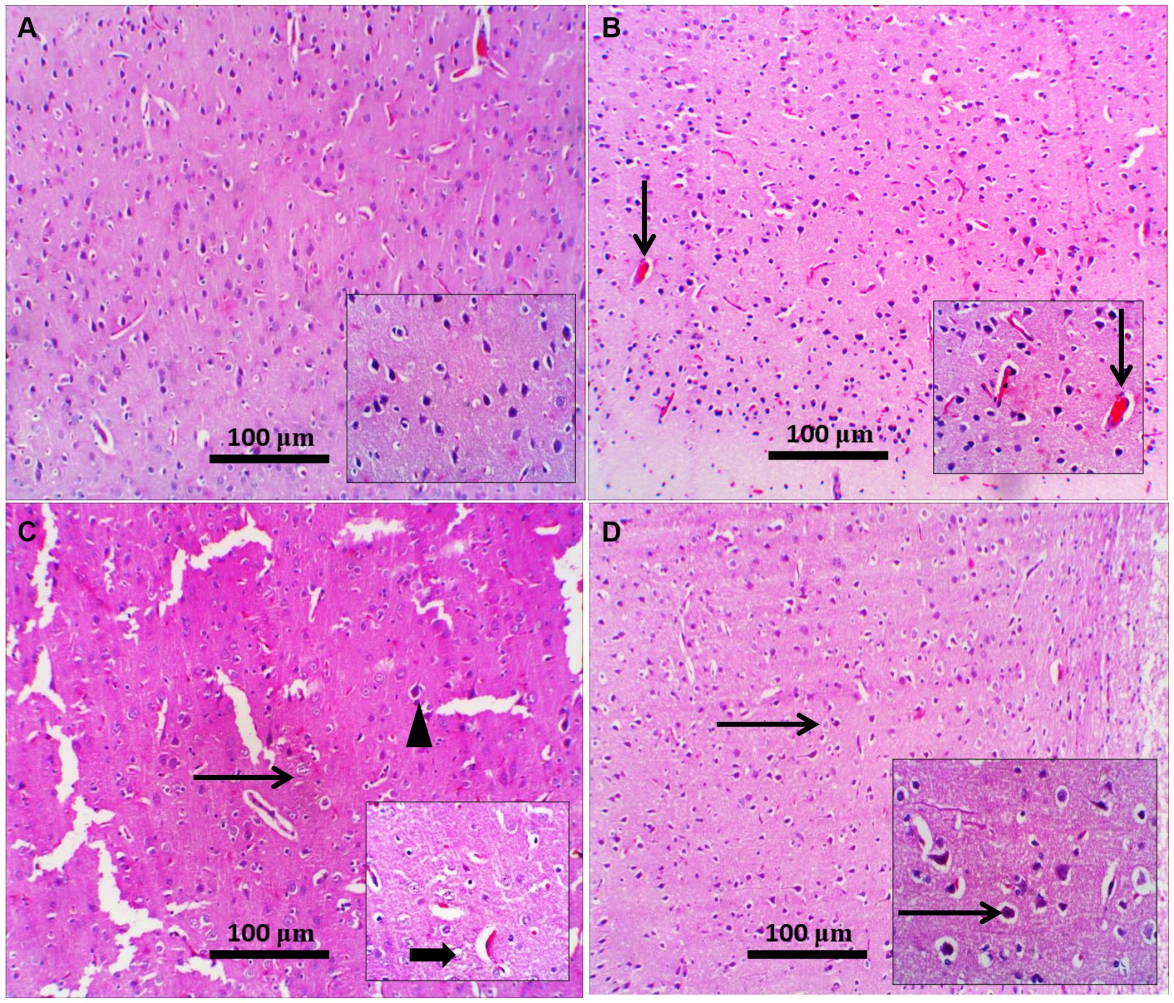
Representative photomicrographs of the prefrontal cortex from different treatment groups. **(A)** Control prefrontal cortex showed normal histological appearance of neuropil and pyramidal neurons; inset demonstrated higher-power pyramidal neurons; occasionally degenerated neurons were present. **(B)** The Zamzam group showed normal pyramidal neurons with a few congested blood vessels (thin arrow; inset). **(C)** The diabetic group showed neuropil rarefaction (increased pallor with loss of normal architecture), intercellular vacuolation (thin arrow), and shrunken pyramidal neurons (arrowhead). Inset: neuronal vacuolation with chromatolysis (thick arrow) and dilated blood vessels. **(D)** Diabetic and Zamzam groups show mild-to-moderate neuronal shrinking with a halo area around it (thin arrows; inset). Mag = 100x, and inset = 400x.

**Figure 8:**
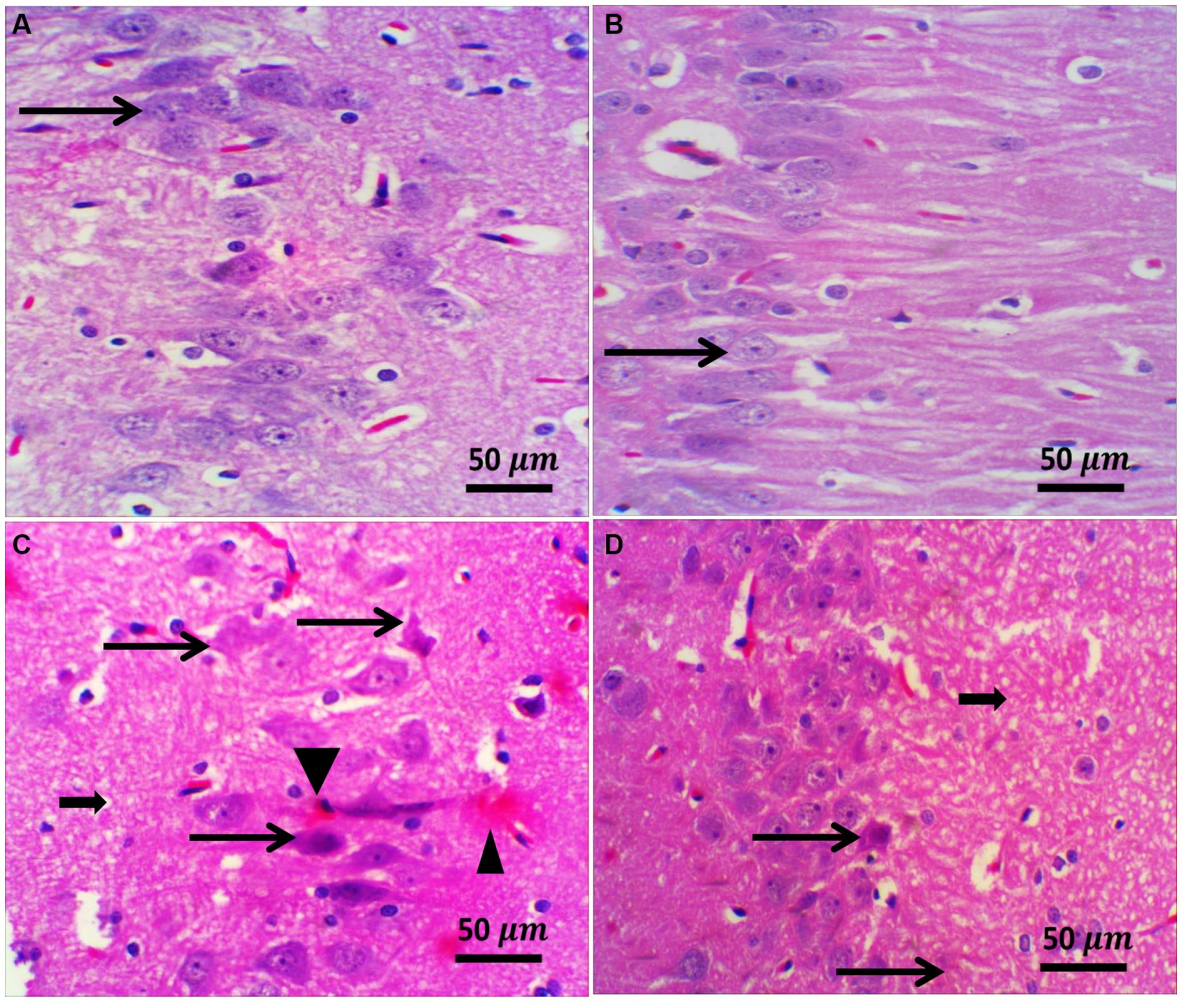
Representative photomicrographs of the hippocampus from different treatment groups. **(A,B)** The control and ZW groups showed normal hippocampal architecture in the CA3 region; pyramidal neurons appeared larger and were arranged in two to five cell layers (thin arrows). **(C)** The diabetic group demonstrated neuronal shrinkage with a faded nucleus (thin arrow) surrounded by glial cells (satellitosis), neuropil vacuolation (thick arrow), and congested blood vessels (arrowhead). **(D)** Diabetic and Zamzam mice showed occasional necrotic neurons (thin arrow) and mild neuropil vacuolation (thick arrow). Magnification = 50x, and inset = 400x.

H&E staining of the diabetic rat prefrontal cortex revealed variable neuronal degeneration in the form of neuropil rarefaction, intercellular vacuolation, shrinking of pyramidal neurons with neuronal vacuolation and chromatolysis, and dilated blood vessels (Figure 7C). The hippocampal C3 region had shrunken neurons with faded nuclei surrounded by glial cells, neuropil vacuolation, and congested blood vessels (Figure 8C). Diabetic rats that drank ZW showed prefrontal sections with mild-to-moderate neuronal shrinking (Figure 7D) and hippocampal sections with mild necrotic neurons and neuropil vacuolation (Figure 8D).

### 3.5 Anti-inflammatory effect of Zamzam water against STZ-induced neuroinflammation

**Figure 9:**
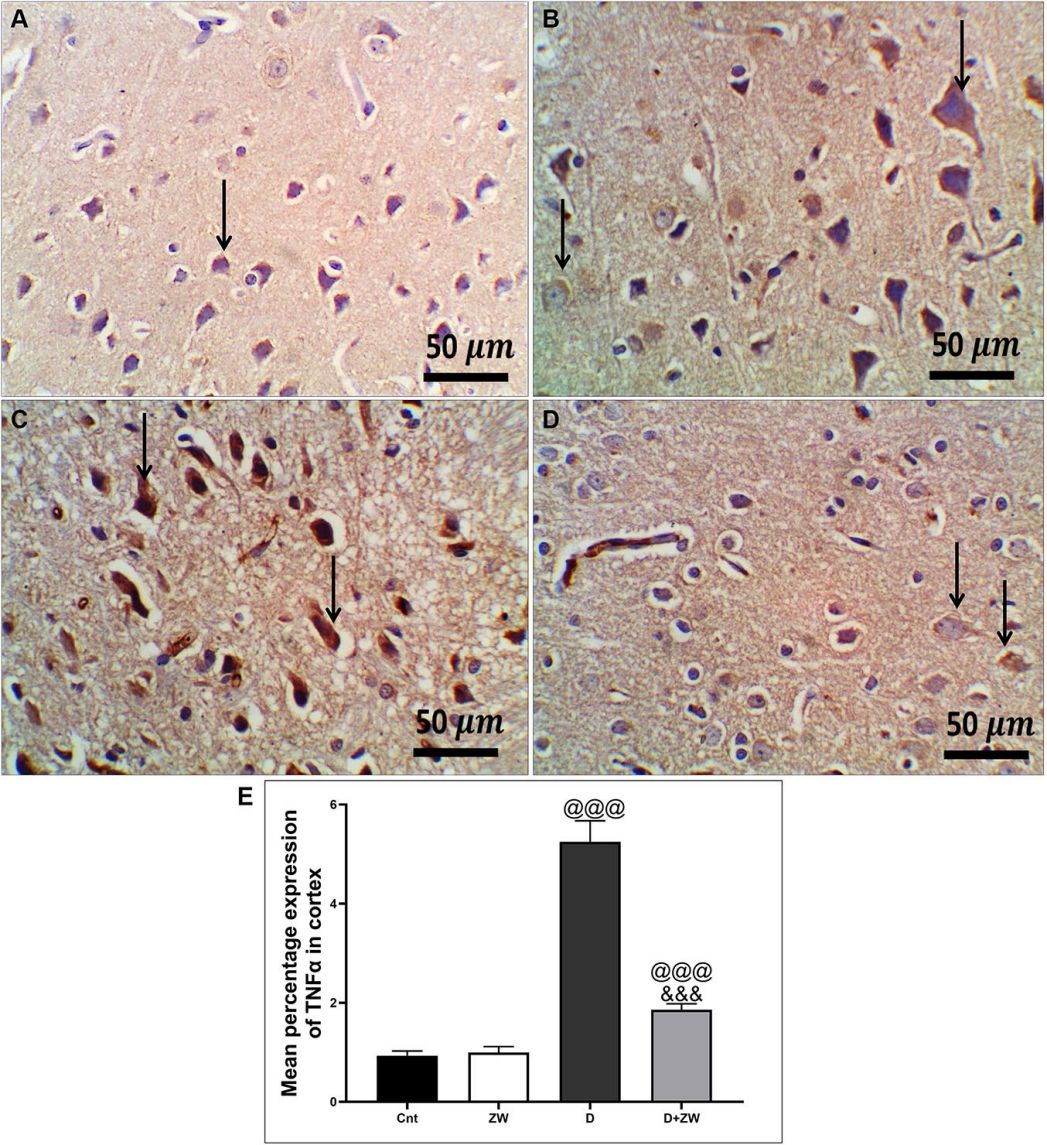
Representative photomicrographs of TNF-α expression in the prefrontal cortex of different treatment groups. **(A)** The cortical region of the control group showed a few faint cytoplasmic expressions in the pyramidal neurons of the cortex. **(B)** The Zamzam group showed minimal cytoplasmic expression in pyramidal neurons and neurological cells of the cortex. **(C)** The diabetic group exhibited strong immunopositive expression of TNF in the cytoplasm of the cortical neurons. **(D)** Diabetic + ZW mice demonstrated mild expression of TNF in the cytoplasm of cortical neuronal cells. Thin arrows indicate immunopositive stained cells. **(E)** Data were expressed as mean ± SD. ^@@^*p* < 0.01, ^@@@^*p* < 0.001 vs. the control group; ^&&&^*p* < 0.001 vs. the diabetic group. Magnification = 50x, and inset = 400x.

**Figure 10:**
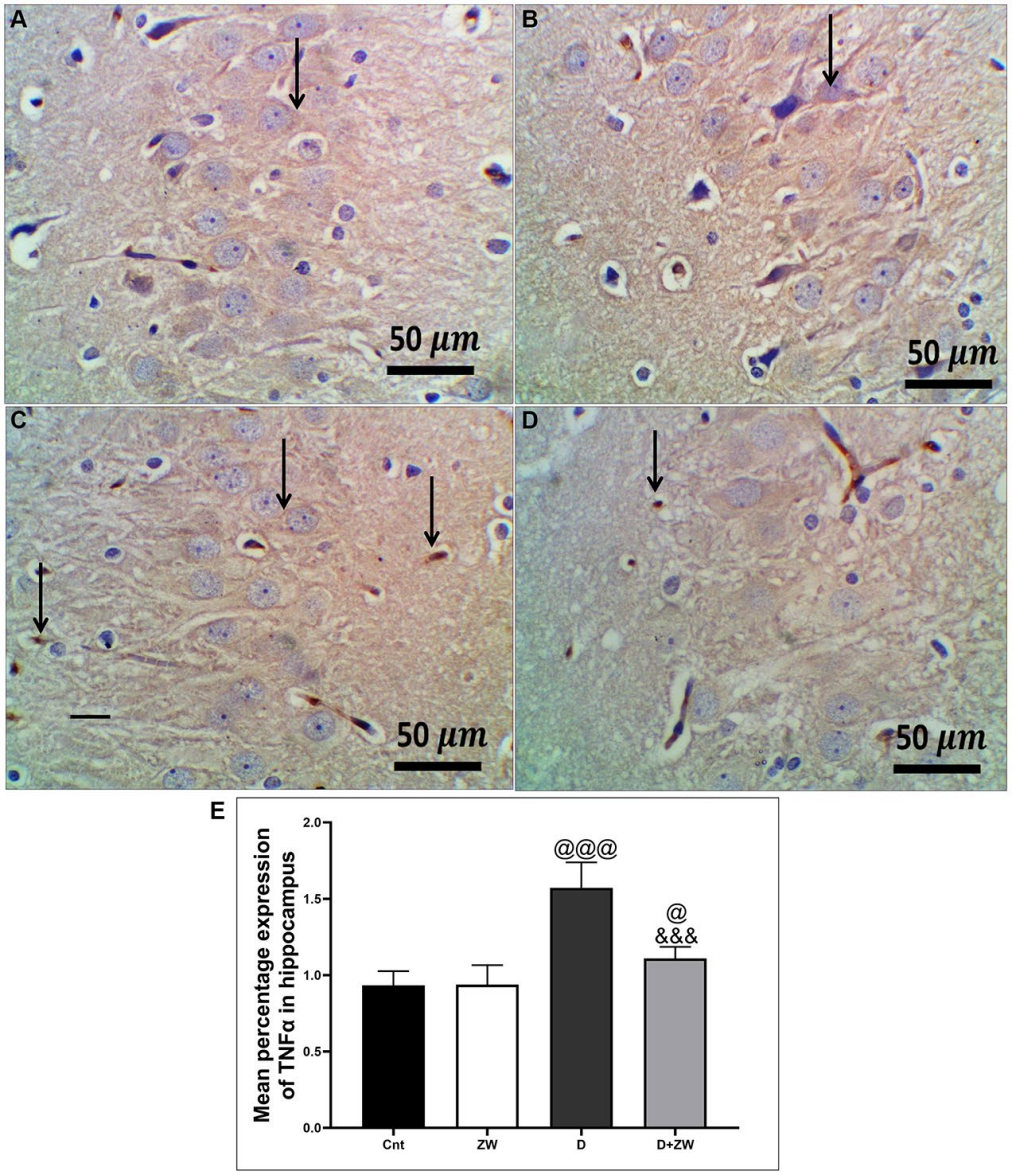
Representative photomicrographs of TNF-α expression in the hippocampal CA3 region in different treatment groups. **(A)** The hippocampus of the control group showed a few faint expressions in hippocampal cells. **(B)** The Zamzam group demonstrated minimal expression in hippocampal neurons. **(C)** The diabetic group showed few faint expressions in the hippocampal neurological cells. **(D)** Diabetic+ ZW mice showing mild, occasional positive immunostained glial cells in the hippocampus. Thin arrow = immunopositivity of stained cells. **(E)** Data were expressed as mean ± SD. ^@@^*p* < 0.01, ^@@@^*p* < 0.001 vs. the control group; ^&&&^*p* < 0.001 vs. the diabetic group. Magnification = 50x, and inset = 400x.

**Figure 11:**
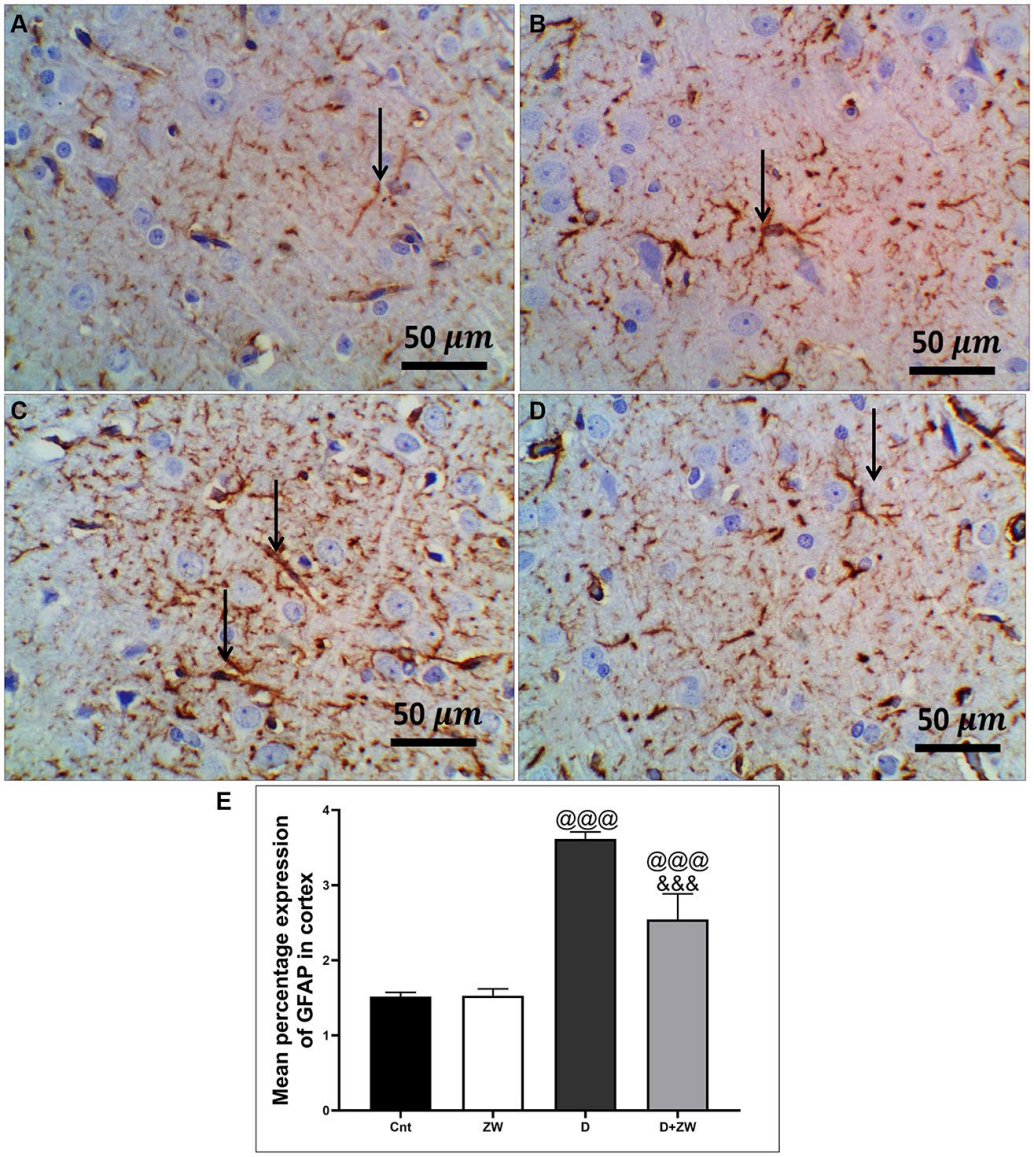
Representative photomicrographs of GFAB expression in the prefrontal cortex of different treatment groups. **(A)** Control group of the cortex region showing minimal immunopositive fibrillar GFAP-stained astrocytic neuropils. **(B)** The Zamzam group showed minimal branching and positive immunostaining. **(C)** The diabetic group demonstrated dense immunopositivity and fibrillar staining of the neurological cells. **(D)** The D + ZW group showed mild to moderately immunopositive expressed fibrillar GFAP in neuropils, astrocytes, and neurological cells. The thin arrows indicate positive immunostaining. **(E)** Data were expressed as mean ± SD. ^@@^*p* < 0.01, ^@@@^*p* < 0.001 vs. the control group; ^&&&^*p* < 0.001 vs. the diabetic group. Magnification = 50x, and inset = 400x.

**Figure 12:**
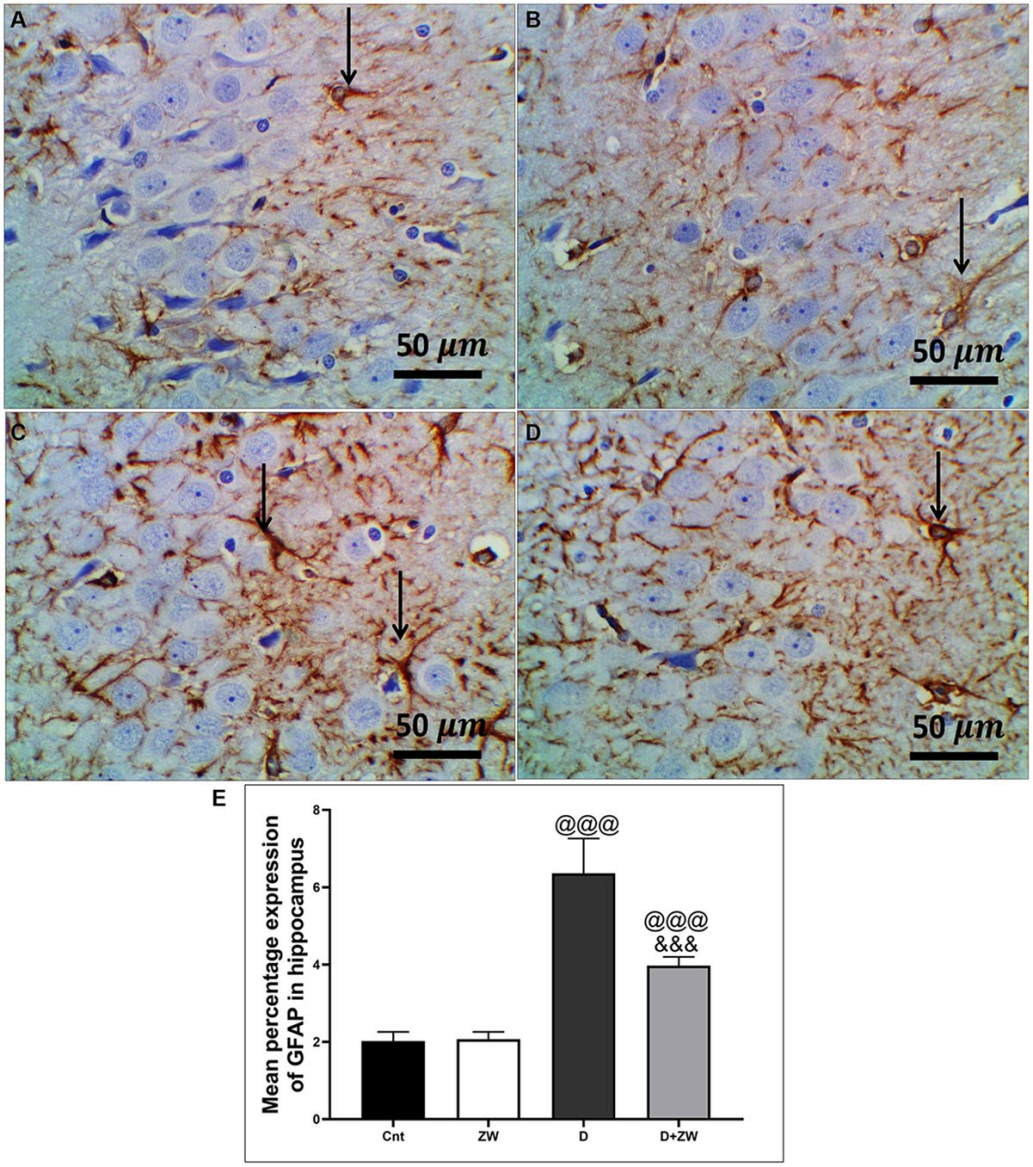
Representative photomicrographs of GFAB expression in the hippocampus region CA3 of the different treatment groups. **(A)** The control group of the hippocampus showed minimal immunopositivity for fibrillar GFAP-stained astrocytic neuropils. **(B)** The Zamzam group showed minimal branching-positive immunostained cells. **(C)** The diabetic group demonstrated dense immunopositive fibrillar staining of the neurological cells. **(D)** The Z + D group showed mild-to-moderate immunopositivity of fibrillar GFAP in the neuropil, astrocytes, and neurological cells. The thin arrows indicated positive immunostaining. **(E)** Data were expressed as mean ± SD. ^@@^*p* < 0.01; ^@@@^*p* < 0.001 vs. the control group; ^&&&^*p* < 0.001 vs. the diabetic group. Magnification = 50x, and inset = 400x.

STZ-induced diabetes exhibited severe neuroinflammation, marked by a significant *p* < 0.001 increase in cortical gene expression of the nuclear transcription factor NfκB, with a subsequent increase in the mRNA levels of the inflammatory cytokines IL-1β and IL6 by 463.2, 508.9, and 368.6%, respectively (Figures 4A,C,E) and immunoexpression of TNF-α by 564.5% (Figures 9C,E) relative to those in control group (Figure 9A). Additionally, the hippocampal mRNA levels of NfκB, IL-1β, and IL6 were 420.8, 497.7, and 343.6%, respectively (Figures 4B,D,F), and the immunoexpression of TNF-α was increased by 168.8% (Figures 10C,E) compared to rats in the control group (Figure 10A). Furthermore, diabetes significantly (*p* < 0.001) increased the number of activated astrocytes by upregulating the immunoexpression of cortical and hippocampal GFAP by 239 and 314.8%, respectively (Figures 11C,E, 12C,E) compared to control rats (Figures 11A,12A, respectively). In contrast, treatment of diabetic rats with ZW significantly (*p* < 0.001) decreased the gene expression of cortical NfκB, IL-1β, and IL6 by 58.4, 55.6, and 55.6%, respectively, and the immunoexpression of TNF-α and GFAP by 35.4 and 70.3 (Figures 9D, 11D), hippocampal gene expression of NfκB, IL-1β, and IL6 by 53.5, 54.8, and 57.4%, respectively, and immunoexpression of TNF-α and GFAP by 70 and 62.2%, respectively (Figures 10D, 12D) groups than in the diabetic group. ZW also significantly (*p* < 0.001) decreases the serum level of TNF-α and CRP by 68.4 and 65.3%, respectively, compared to diabetic rats (Figures 4I,J). These results suggested that ZW possesses strong anti-inflammatory activity against diabetes-induced neuroinflammation.

### 3.6 Impact of Zamzam water on cortical and hippocampal neurotransmitters

**Figure 13:**
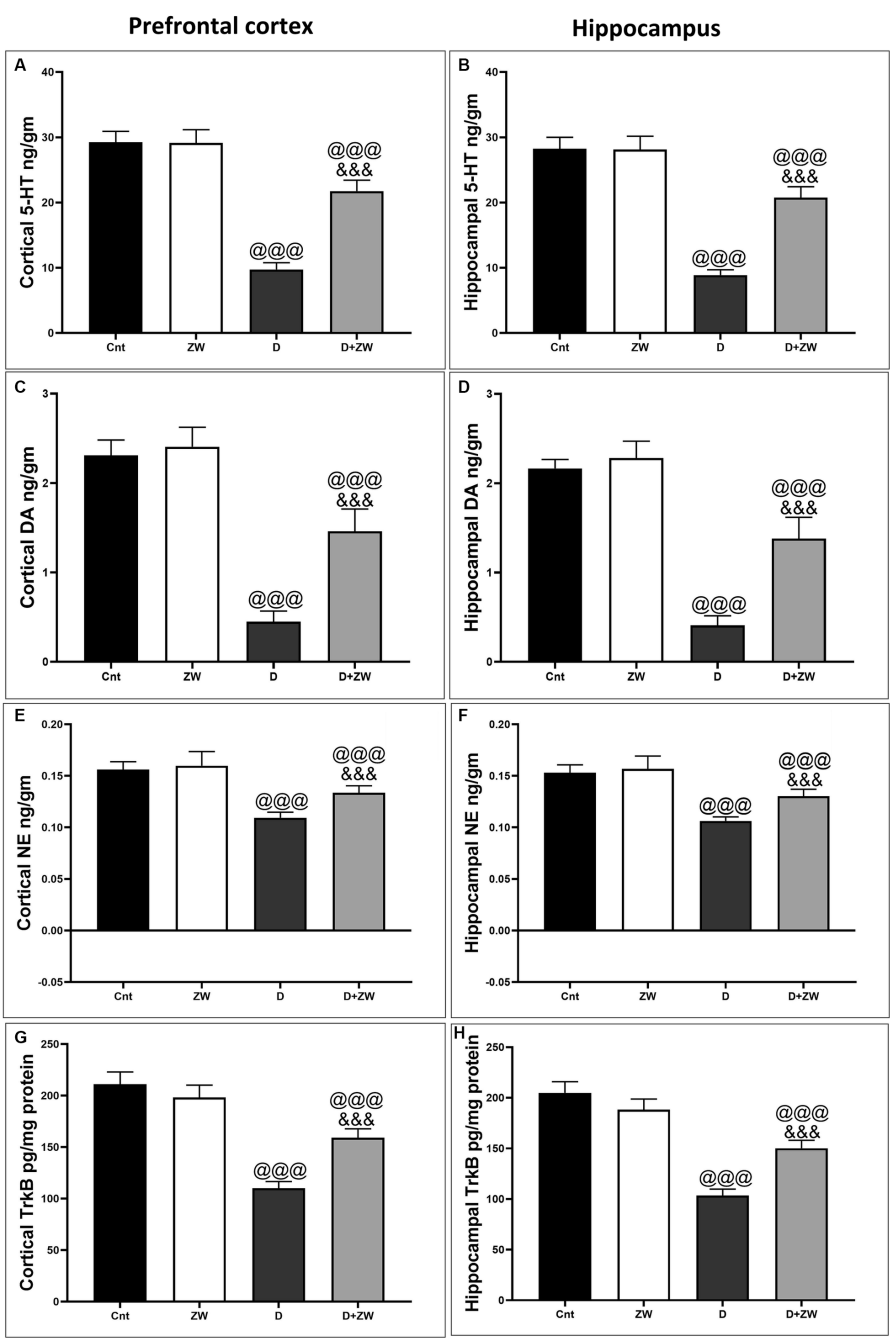
Effects of ZW on protein expression in the prefrontal cortex and hippocampus. **(A,B)** 5-HT, **(C,D)** dopamine, **(E,F)** norepinephrine, and **(G,H)** TrkB levels. Data were expressed as mean ± SD. ^@@@^*p* < 0.001 vs. the control group; ^&&&^*p* < 0.001 vs. the diabetic group.

As shown in Figure 13, diabetic rats that received ZW showed a significant increase (*p* < 0.001) in the cortical levels of the monoamine neurotransmitters serotonin (5-HT), dopamine (DA), and norepinephrine (NE) by 222.5, 324.4, and 122%, respectively (Figures 13A,C,E), as well as hippocampal 5-HT, DA, and NE by 233.9, 345, and 122.6%, respectively, compared with the diabetic group (Figures 13B,D,F). This finding explored the upregulatory effect of ZW on monoamine neurotransmitters in the prefrontal cortex and hippocampus of diabetic rats.

### 3.7 Impact of Zamzam water on BDNF/ERK/CREP pathway in the prefrontal cortex and CA3 hippocampal region of diabetic rats

**Figure 14:**
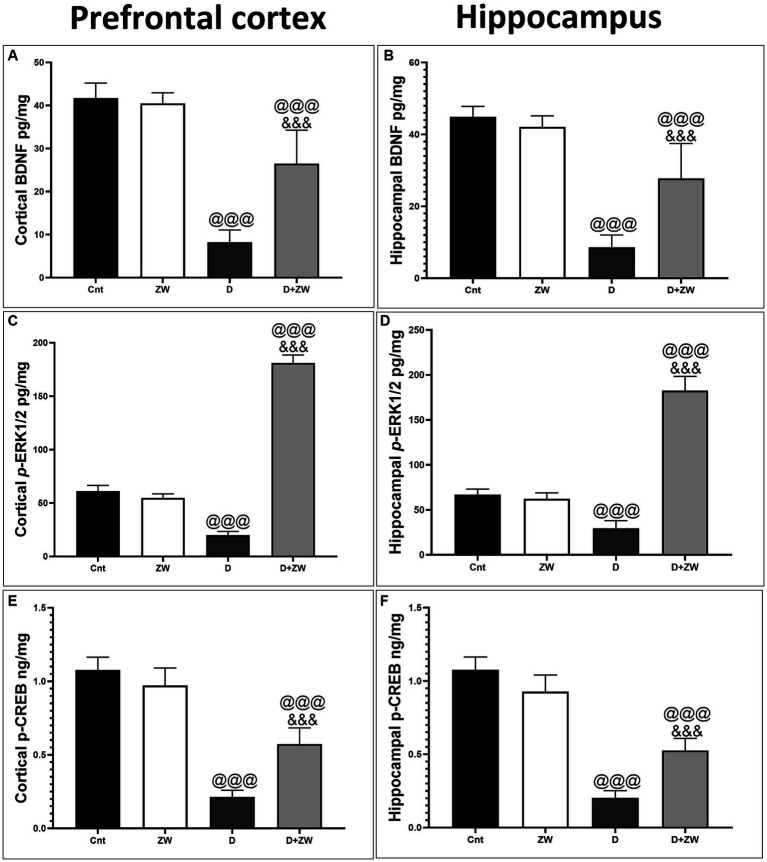
Effect of ZW on protein expression in the prefrontal cortex and hippocampus **(A,B)** BDNF **(C,D)** p-ERK1 **(E,F)** p-CREP. Data were expressed as mean ± SD. ^@@@^*p* < 0.001 vs. the control group; ^&&&^*p* < 0.001 vs. the diabetic group.

As shown in Figure 14, the prefrontal cortex and hippocampus of diabetic rats showed a significant *p* < 0.001 depression in the protein levels of BDNF by 50.6 and 52% (Figures 14A,B), p-ERK1/2 by 30.8 and 22.5% (Figures 14C,D), and p-CREP by 19.6 and 18.6% (Figures 14E,F), respectively, compared to control rats. Interestingly, drinking ZW in diabetic rats significantly (*p* < 0.001) increased the mRNA expression of BDNF by 321 and 322%, p-ERK1/2 by 910 and 614%, and p-CREP by 271.4 and 260%, respectively. This result indicated that ZW upregulated the cortical and hippocampal BDNF/ERK/CREP pathways.

### 3.8 Ameliorative effect of Zamzam water on diabetic-induced neuronal apoptosis

Diabetic rats induced significant *p* < 0.001 neuronal apoptosis by increasing the number of caspase-3 immunostained positive apoptotic cells in the prefrontal cortex and hippocampus by 283.6 and 213.2%, respectively, compared to control rats (Supplementary Figures 15C, 16C). Diabetic rats that were administered ZW demonstrated a notable reduction (*p* < 0.001) in the number of caspase-3-positive cells in both the prefrontal cortex and hippocampus when compared to untreated diabetic rats. The reductions were 56.3 and 38.6% in each area, respectively (Supplementary Figures 15D, 16D). The inhibitory effect of ZW on diabetes-induced neuronal apoptosis highlights its ameliorative effect on neuronal histology.

## 4 Discussion

Many patients with diabetes suffer from neurological dysfunction (Kodl and Seaquist, 2008). Patients with diabetes suffer from two common behavioral disorders: anxiety and depression (Collins et al., 2009). One of the most common psychological disorders is anxiety, which appears to be more morbid and interferes with lifestyle when it is associated with depression (Vogelzangs et al., 2013). Previous research has shown that anxiety symptoms increase in patients with diabetes (Engum, 2007; Smith et al., 2013). The prevalence of depression in type 2 diabetes patients is twice that in healthy individuals (Siddiqui, 2014). Several factors are involved in the pathogenesis of chronic elevated blood sugar-induced depression, such as dysregulation of the hypothalamic–pituitary–adrenal axis and immune system, depression in the levels of brain monoamines, neuronal degeneration, impaired synaptic plasticity, and oxidative insult (da Silva Haeser et al., 2007). Furthermore, neuroinflammation and depression are strongly associated (Stuart and Baune, 2012).

The main findings of our study reported that ZW, a well-known alkaline water, improved depression-like behavior by decreasing the immobility time in the FST and anxiety-like behavior, as indicated by the elevated percentage of open field entry and time in the EPMT. Furthermore, it increased center crossing and distance traveled, with a decrease in grooming time and the number of fecal boils in the OFT. Moreover, ZW enhanced the activities of SOD, CAT, GSH, and total antioxidant capacity, with preserved histological structures in the prefrontal cortex and hippocampus. Additionally, it enhanced the HPA axis and depressed cortical and hippocampal NFκB, TNF-α, IL-1β, IL6, GFAP, and serum level of TNF-α and CRP, with upregulation of the BDNF/ERK/CREP pathway and 5-HT, NE, and DA monoamine neurotransmitters. It also improved neuronal survival and decreased caspase-3-stained apoptotic cells.

The diabetic rat group demonstrated behavioral disorder as the immobility time significantly increased in the FST, which is considered a common depression-like behavior test, but there was no significant difference between the different experimental groups, side by side, in diabetes-induced depression-like behavior. In addition, diabetic rats showed anxiety-like behavior in the form of decreased OAE% and OAT% in the EPMT, as well as decreased time spent in the central zone and traveling distance, while increasing grooming time and fecal boli in the OFT. In agreement with our behavioral findings, previous studies by Gomez et al. (2001) documented that STZ-induced diabetes rats exhibited depression-like behavior in the FST, and Aksu et al. (2012) reported that diabetic rats stayed in the central zone for a short time and stayed in closed arms in the EPMT. In contrast, diabetic rats that received ZW showed anxiolytic-like behavior by decreasing immobility time in the FST and antidepressant-like behavior by increasing OAE% and OAT% in the EPM test, as well as increasing the time spent in the center zone by decreasing grooming time and fecal boli. ZW had a positive effect on despair behavior associated with the prefrontal cortex and C3 hippocampal region morphological improvement in the form of fewer necrotic neurons and mild neuropil vacuolation. Control rats that received ZW showed no significant changes in the depressive and anxiety-like behavior tests.

Several mechanisms are involved in the ameliorative effects of ZW on STZ-induced depressive and anxiety-like behaviors. Hyperglycemia leads to the accumulation of reactive oxygen species that consume antioxidant enzymes (Maritim et al., 2003). Oxidative insults have been shown to play a leading role in depression and anxiety pathophysiology (Palta et al., 2014). In this study, homogenates of the prefrontal cortex and hippocampus of diabetic rats showed a significant decrease in SOD, CAT, GSH, and antioxidant enzyme levels. ZW exerts antioxidant activity against cortical and hippocampal oxidative stress by significantly increasing the activity of antioxidant enzymes centrally and serum level of total antioxidant capacity peripherally. The neural antioxidant power of ZW is thought to be due to its abundance of trace elements involved in the synthesis of antioxidant enzymes, as well as its upregulating effect on the Nrf2/HO-1 pathway, a nuclear gene regulator for antioxidant enzyme synthesis. Our results corroborated those of our previous study by Taha et al. (2023b), which documented the upregulation of antioxidant enzymes and Nrf2/HO-1 in gentamicin-induced testicular oxidative stress. Bamosa et al. (2013) reported a protective effect of ZW against diabetes-induced oxidative stress by increasing the levels of antioxidant enzymes.

Diabetes not only impairs antioxidant integrity but also initiates neuroinflammatory reactions by activating the nuclear transcription factor NfκB, which stimulates IL6, IL-1β, and TNF-α pro-inflammatory cytokines (Gloire et al., 2006). One of these inflammatory mediators, TNF-α, can cross the blood–brain barrier (Nishioku et al., 2010) and stimulate astrocyte activation, thereby increasing the production of inflammatory cytokines (Baune et al., 2012). Neuroinflammation plays a crucial role in the pathogenesis of hyperglycemia-induced anxiety and depression (Zhou et al., 2018). Moreover, the pro-inflammatory mediators IL-6, IL-1β, and TNF-α are elevated in diabetes-induced anxiety and depression (Aswar et al., 2017). In our study, diabetes elevated the prefrontal cortex and hippocampal gene expression of NfκB, IL-1β, and IL6, as well as the immunoexpression of TNF-α and GFAP, which are considered indicators of astrocyte activation. Interestingly, ZW inhibited diabetes-induced anxiety and depression by minimizing the neuroinflammatory response by decreasing NfκB, IL-1β, and IL6 mRNA levels and the number of TNF-α- and GFAP-immunostained neurons and serum level of TNF-α and CRP because of its antioxidant power. Consistent with our findings, a previous study was conducted by Taha et al. (2023b), who documented the anti-inflammatory effects of ZW against gentamicin-induced testicular toxicity by decreasing NfκB immunoexpression and the protein levels of pro-inflammatory cytokines TNF-α, IL-1β, and IL6. Additionally, Moni et al. (2022) conducted a study that demonstrated that ZW can reduce the concentration of pro-inflammatory cytokines in normal wounds, which is attributed to its zinc components that provide anti-inflammatory benefits.

Neuroinflammation impairs neurogenesis and overstimulates the hypothalamic–pituitary–adrenal (HPA) axis, which is considered one of the principal mechanisms in the pathophysiology of depression (Pariante and Lightman, 2008). A continuous increase in cortisol levels results in hippocampal neuronal damage (McAllister-Williams et al., 1998). Treatment of diabetic rats with ZW significantly decreased serum cortisol levels compared with those in diabetic rats. The downregulatory effect of ZW on plasma cortisol levels can be attributed to its high zinc content, which is thought to normalize cortisol levels and subsequently ameliorate depressive symptoms in humans and rodents (Tassabehji et al., 2008; Solati et al., 2015). Zinc is the second-most abundant metal in the brain, after iron (Huang, 1997). Decreased levels of plasma cortisol induced by ZW exhibit negative feedback on the HPA axis, with subsequent elevations in the serum levels of ACTH and depressions in CRH secretion.

Several studies have shown that 5-HT, NE, and DA neurotransmitters play crucial roles in mood regulation, stress response motivation, and cognitive performance (Gu et al., 2018). Studies have reported that 5-HT plays a major role in the pathogenesis of depression (Nemeroff and Owens, 2009). NE deficiency is also a risk factor for depression (Schildkraut, 1967). Dopamine deficiency is also linked to depression (Friedman et al., 2014). Neuroinflammation and oxidative insults explain the increase in monoamine oxidase transcription (Meulendyke et al., 2014). In this study, diabetic rats drinking ZW showed a significant elevation in the levels of the prefrontal cortex and hippocampal monoamine neurotransmitters compared to those in the diabetic group. The upregulation of monoamine neurotransmitters by ZW is due to its powerful antioxidant and anti-inflammatory effects, and zinc-dependent proteins (GPR39) have been linked to serotonin synthesis (Tena-Campos et al., 2015).

BDNF is one of the most common neurotrophic factors in the central nervous system that suppresses depression-like behaviors (Huang et al., 2011). Its functions can be summarized as neurogenesis regulation, synaptic plasticity, and the release of neurotransmitters (Bae et al., 2016). Moreover, it is essential for the development, growth, and differentiation of neuronal cells to prevent apoptosis (Zhang et al., 2016). Several antidepressant drugs act on BDNF and its receptor Trkβ to improve hippocampal neurogenesis (Ho et al., 2013). Zhang et al. (2016) showed that the loss of BDNF and its receptor leads to hippocampal neuronal atrophy and apoptosis, resulting in depression-like behavior. The binding of BDNF to its specific receptor activates the BDNF/ERK/CREP pathway and regulates synaptic plasticity (Jiang et al., 2012; Yi et al., 2014). ERK signaling, including ERK1 and ERK2, plays a role in the response of neuronal cells to external stimuli and is activated by BDNF (Autry and Monteggia, 2012). ERK activation successfully activates CREP phosphorylation, which plays a critical role in synaptic plasticity, new synapse formation, and neuronal survival and exhibits an antidepressant effect (Agosto-Marlin and Mitchell, 2017). In the present study, the diabetic groups showed significant downregulation in the protein levels of the BDNF/ERK/CREP pathway components in the prefrontal cortex and CA3 hippocampal region, as well as a decrease in the protein level of the BDNF receptor Trkβ.

In contrast, drinking ZW in diabetic rats significantly enhanced the protein expression of the BDNF/ERK/CREP pathway and its receptor Trkβ. These findings are in line with those of a previous study by Ali et al. (2009), who documented the upregulatory effect of ZW on the level of BDNF in the endometrium, helping uterine flashing. In addition, the excitatory role of ZW in neurogenesis and synaptic plasticity is attributed to its high zinc content, which plays a major role in newly formed brain cells (Gao et al., 2009) and enhances synaptic plasticity (Pfaender et al., 2016). Similarly, the up-regulatory effect of ZW on the BDNF/ERK/CREP pathway was associated with a significant decrease in caspase-3 apoptotic neuronal cells in the prefrontal cortex and CA3 hippocampal region concerning the diabetic group, which explained the histological improvement with H&E staining and was reflected in the improvement in neuronal despair.

## 5 Conclusion

In brief, our findings provided insight into the beneficial effects of ZW against diabetes-induced anxiety and depression-like effects. ZW improved the cortical and C3 hippocampal morphology, modulating the HPA axis with an increase in the protein levels of 5-HT, DA, and NE monoamines. In addition, ZW elevated antioxidant enzyme levels and upregulated the Nrf2/HO-1 pathway. Moreover, it inhibited diabetes-induced neuroinflammation by decreasing the inflammatory markers NfκB, TNF-α, IL-1β, and IL-6 and downregulating the BDNF/ERK/CREP pathway.

## Data availability statement

The original contributions presented in the study are included in the article/Supplementary material, further inquiries can be directed to the corresponding author.

## Ethics statement

The animal studies were approved by Mansoura University Animal Care and Use Committee with a code number (Code No: MU-AUCU: VM.R.22.12.37). The studies were conducted in accordance with the local legislation and institutional requirements. Written informed consent was obtained from the owners for the participation of their animals in this study.

## Author contributions

MT: Conceptualization, Writing – original draft. MM: Investigation, Writing – review & editing. AA-K: Data curation, Writing – original draft. AS: Conceptualization, Writing – review & editing. OA: Methodology, Writing – original draft. TB: Supervision, Writing – original draft. OB: Writing – review & editing. IE-S: Data curation, Writing – review & editing. NQ: Methodology, Writing – original draft. SE: Methodology, Writing - review & editing.
