# Supplementary material for: Anxiolytic and antidepressant like effects of Zamzam water in STZ-induced diabetic rats, targeting oxidative stress, neuroinflammation, BDNF/ERK/CREP pathway with modulation of hypothalamo-pituitary–adrenal axis
Source: Front Neurosci. 2023 Dec 1;17:1265134. doi: 10.3389/fnins.2023.1265134 (PMC10722298; doi:10.3389/fnins.2023.1265134)
Supplement: Supplementary file 1 [file Table_1.DOCX]

Supplementary Material

**Supplementary FIGURE 15.** Representative photomicrographs of caspase-3 expression in the prefrontal cortex of different treatment groups. **(A, B)** The cortex region of the control group showed mild immunopositive caspase expression in cortical neurons and low expression in glial cells. **(B)** The Zamzam group showed low cytoplasmic caspase expression in neurons, with few immunopositive cytoplasmic and nuclear-stained neurons. **(C)** The diabetic group showed moderate caspase expression in cortical neuronal cells. **(D)** The Z+D group exhibited faint caspase expression in the cytoplasm of neurons, with a few immunopositive stained neurological cells. The blue arrow indicates nuclear and cytoplasmic expression in glial cells, and the black arrowhead indicates cytoplasmic expression. **(E)** Data were expressed as the mean ± SD. ^@@^ p < 0.01, ^@@@^ p < 0.001 vs. the control group; ^&&&^ p < 0.001 vs. the diabetic group. Magnification = 50x, and inset = 400x.

**Supplementary FIGURE 16.** Representative photomicrographs of caspase-3 expression in the hippocampus region CA3 in the different treatment groups. **(A)** The hippocampus of the control group exhibited mild immunopositive caspase expression. **(B)** The Zamzam group showed low cytoplasmic expression of caspase in neurons, with few immunopositive cytoplasmic and nuclear-stained neurological cells. **(C)** Diabetic groups showed moderate expression of caspases in the neurons and neurological cells of the cortex. **(D)** The Z+D group showed faint caspase expression in the cytoplasm of neurons, with few immunopositive stained neurological cells. Arrow = nuclear and cytoplasmic expression in glial cells; black arrowhead indicates cytoplasmic expression. **(E)** Data were expressed as mean ± SD. ^@@^ p < 0.01, ^@@@^ p < 0.001 vs. the control group; ^&&&^ p < 0.001 vs. the diabetic group. Magnification = 50x, and inset = 400x.
