# Supplementary figures and images for: Anxiolytic and antidepressant like effects of Zamzam water in STZ-induced diabetic rats, targeting oxidative stress, neuroinflammation, BDNF/ERK/CREP pathway with modulation of hypothalamo-pituitary–adrenal axis
Source: Front Neurosci. 2023 Dec 1;17:1265134. doi: 10.3389/fnins.2023.1265134 (PMC10722298; doi:10.3389/fnins.2023.1265134)

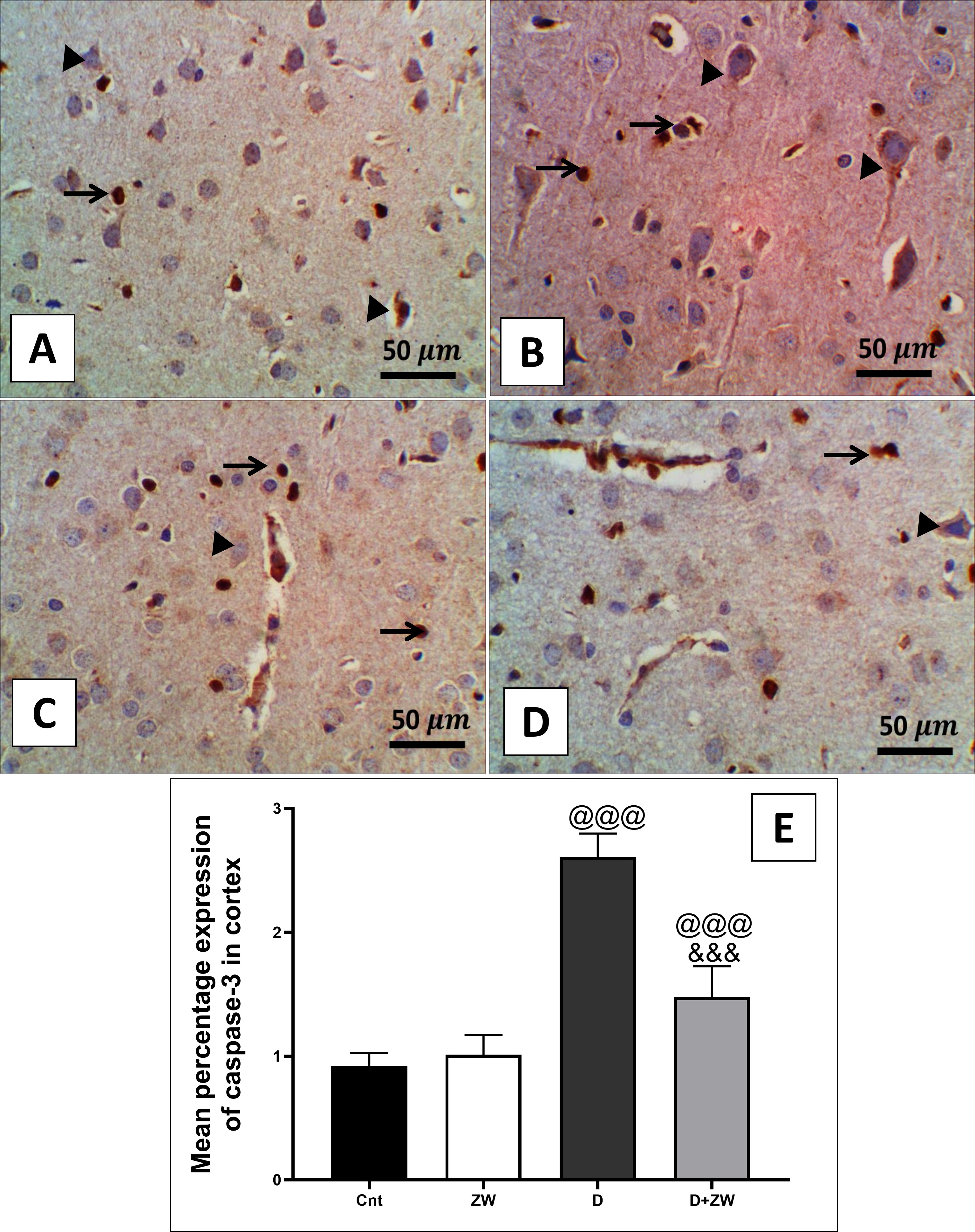

Supplement: Supplementary file 2 [file Image_1.JPEG]

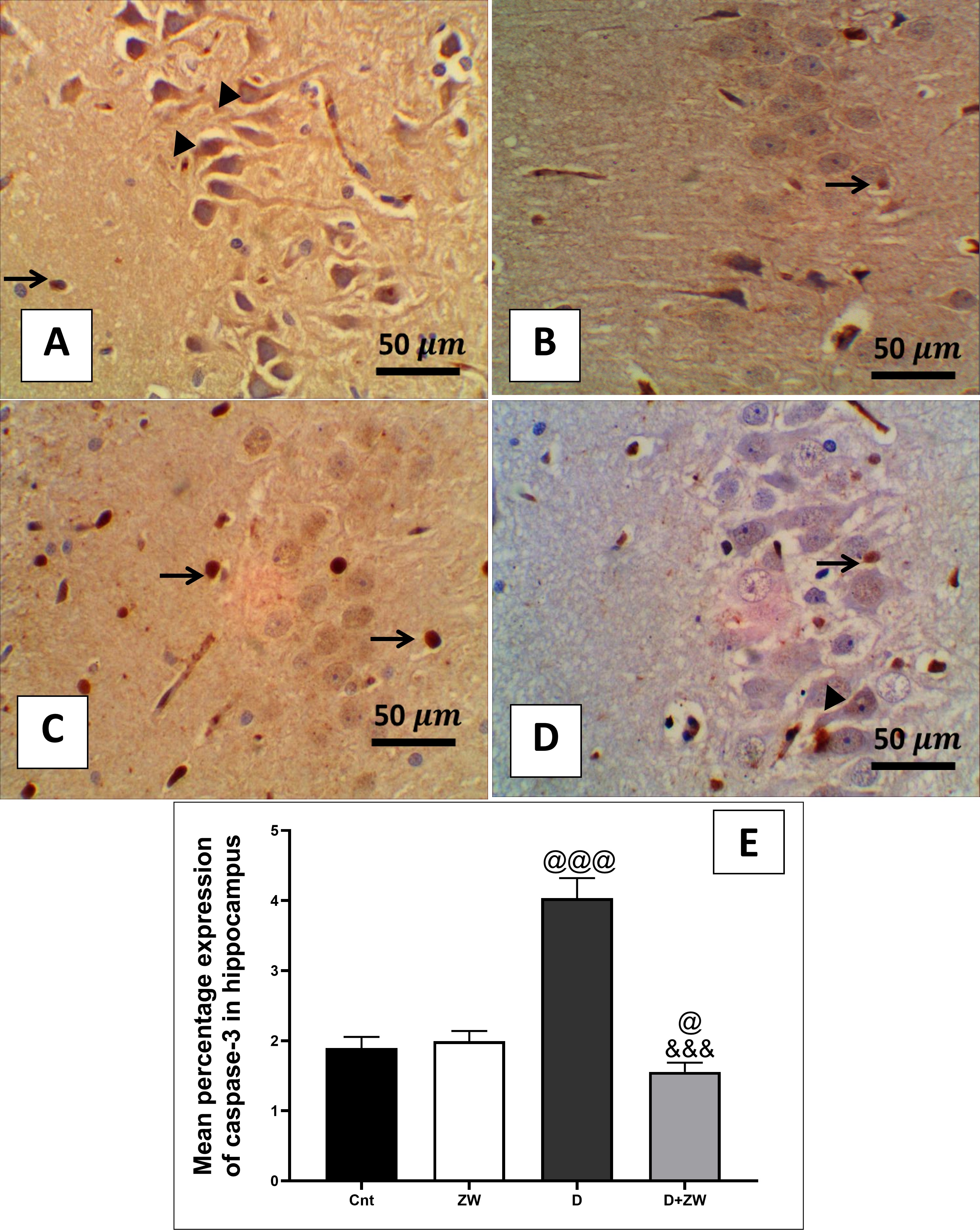

Supplement: Supplementary file 3 [file Image_2.jpg]
